# Uterine Hemorrhage

**Published:** 1873-05

**Authors:** John H. Weir

**Affiliations:** Edwardsville, Illinois


### CORRESPONDENCE.

### UTERINE HEMORRHAGE.

EDWARDSVILLE, ILLINOIS, April 3, 1873.

*Editors Southern Medical Record:*

Will you grant me a small space in your excellent journal, devoted to troublesome, and at times alarming, cases of uterine hemorrhage, under the following heads, viz.:

1st. During labor or parturition;

2d. After labor, and before removing the placenta;

3d. After the placenta has been removed;

4th. Hemorrhage coming on during the month of confinement;

5th. Hemorrhage with woman at other times than child-bearing periods—at and subsequent to menstruation.

Hemorrhage during labor is very common, but, when excessive, constitutes a good reason for alarm, both to patient and physician. All such profuse bleeding, in my judgment, is caused from a portion of the placenta being torn from the attachment to the uterus, either complete or partial placenta previa, or higher up by injuries or irregular uterine contractions. Profuse hemorrhage during labor always produces great relaxation and dilatation of the os uteri, and speedy delivery of the child—the best if not the only remedy. In this exhausted condition from loss of blood, though the expulsive pains are feeble, the resistance to the passage of the child must be also comparatively feeble, and would always advise, in such cases, the rupturing of the amnion, so as to hasten delivery. The descent of the child into the cavity of the pelvis will almost, if not completely, stop the flow of blood, and give time to rally. When a partial or complete placenta previa exists, after rupturing the amnion, the bleeding edge of the placenta may be compressed by the physician's fingers until the child has descended sufficient to stop the hemorrhage. Fld. extract of ergot may be given in drachm doses, to increase expulsive contractions of the uterus, if pains are too feeble or slow.

Hemorrhage after delivery of child, and previous to removal of placenta, is frequently met with, and is relieved, in my experience, only by removing the placenta, which, if it does not descend by a gentle draw on the cord, and bleeding continues, the hand should be introduced, following the cord, held firmly by the other hand, the placenta firmly grasped, the hand at the cord be removed to the abdominal tumor and gently but firmly pressed down, when the hand with placenta enclosed will be expelled without further difficulty. If bleeding continues after removal of placenta, whether bleeding has occurred before or after removal, the remedy is easy and simple. Have the patient lie on her back, her knees elevated and separated; stand or sit on her left side, introduce the four fingers of the left hand up to the os uteri, the second and third fingers resting against or slightly within that organ, the first and fourth fingers a little elevated, so as to form a good aqueduct; back of hand thus adjusted pressing down firmly on the perinæum, the thumb of same hand be used as an adjuster of the soft parts above, and keep the passage open, and with the right hand take a pitcher or cup of cold water, pour a small continuous stream into the hand, thus forming a conductor to the os uteri, and into it, if open, and continue till hemorrhage ceases. I have never failed of success in this way, however profuse the bleeding, in a few minutes, for about thirty years, about which time I conceived the above plan of treatment.

In periodical hemorrhage after menstruation, or somehow connected with that function, returning in two, three or four weeks, and continuing from five to ten days more or less profuse, is a difficulty with which many females are afflicted, and more those who do not than those who do bear children. This constant drain, whatever its cause, is so debilitating as to prevent conception, or if it barely takes place, the fetus perishes in embryo, and the result is, a mole or false conception grows as a fungus for two, three or four months, and is then expelled, leaving the unfortunate and disappointed woman in a worse condition than before.

The most effectual remedy I have ever found for the above is the following: Take cotton wool about one-fourth ounce, saturate it thoroughly with glycerin—it will take up about one ounce of glycerin; introduce it up to the os tincæ; let it remain twenty-four hours, and, if necessary, repeat in a day or so. The affinity glycerin has for water will cause a great flow of that liquid, so that it will oblige the patient to wear a napkin. This drain will relieve all congestion of the organs from a livid red to a pale pink, and hemorrhage will disappear.

I have never found this fail to relieve, either in hemorrhage uteri connected with menstruation, or in cases of lying-in women during the month of confinement.

I now propose to give a single illustration of each ailment referred to above, with treatment.

Some years since, I was called to go five miles in the country to see Mrs. M., in labor—her third confinement—healthy, and of full habit. When entering the chamber, found the floor covered with blood, which had passed through the bed and run across the room some ten feet; found the woman in a fainting condition, of pale and bloodless appearance, and pulseless. I instantly made examination—found her lying in a pool of blood—full one-half the placenta was within the vagina, and bleeding freely—found the os uteri fully dilated with amnion entire—passed my hand into the womb, ruptured the membrane, came in contact with the child's head, but believing I could not wait for the tedious head delivery, passed my hand by the head and body of child, grasped its feet, turned it, delivered the woman, I think, within two minutes from the introduction of my hand, and in the absence of any expulsive pain, or even the woman being conscious of what was going on. The placenta followed the child; the woman aroused, bleeding ceased, and child as well as mother are alive and well now. This was a case of complete placenta previa, and, from after-examination, I believe the woman lost near two gallons of blood. I have often congratulated myself for having performed the only thing that could have saved mother or child.

Some time since, I was called by Dr. A. to see Mrs. H., who was in labor and bleeding freely. Her appearance was pale and bloodless, pulse feeble, uterine contractions also feeble. On examination, found os uteri dilated, and felt the edge of placenta, from whence came the bleeding; amnion entire, though somewhat distended by each feeble contraction. I at once ruptured the amnion, compressed the bleeding fragment of the placenta, the child's head passed readily down, and in ten or fifteen minutes the woman was safely delivered of a son, and no further bleeding occurred.

This was a case of partial placenta previa—the placenta came away, and mother and child well.

Where there is so much bleeding, it so exhausts the strength, the uterine contractions are not sufficient to rupture the sac, but when done, relaxation being so complete, makes no resistance, and child passes readily down.

Was called to attend Mrs. C. She was delivered of a healthy child in about the usual time, placenta was removed, and everything seemed in good order. In a few minutes I observed the woman gasping for breath—had a pale, bloodless face. I examined, found she was flooding freely, and nearly pulseless. She became insensible to surrounding objects. I called for water, introduced my left hand as described above, poured in a stream, and in five minutes bleeding ceased and did not return.

Was called to see Mrs. H., who had been confined some four weeks previous; had troublesome uterine hemorrhage for the past week or ten days; her physician had failed to relieve her. I introduced the glycerin in cotton as described above—bleeding ceased and did not return.

Mrs. C., a small, feeble lady, had given birth to three children; the second a son now eight years old—first and third did not live; had been in poor health five or six years; had uterine hemorrhage two-thirds of the time; had not had a child for six years, but had passed two moles at different times; nothing seemed to relieve her almost constant uterine drain. About one year ago I applied the glycerin as described; bleeding stopped; she conceived, and was delivered of a son some two months since.

JONH H. WEIR, M. D.

